# A multidisciplinary approach to assess recovery of consciousness in a patient with moyamoya disease

**DOI:** 10.1002/brb3.1241

**Published:** 2019-04-05

**Authors:** Caterina Formica, Francesco Corallo, Rosa Morabito, Cettina Allone, Simona De Salvo, Katia Micchia, Federica Corallo, Antonino Todaro, Silvia Marino

**Affiliations:** ^1^ Neuroimaging Laboratory IRCCS Centro Neurolesi “Bonino Pulejo” Messina Italy; ^2^ Biomedical Department of Internal Medicine and Medical Specialties University of Palermo Palermo Italy; ^3^ Department of Biomedical and Dental Sciences and Morphological and Functional Imaging University of Messina Messina Italy

**Keywords:** disorders of consciousness, moyamoya disease, neuropsychological evaluation, rehabilitation

## Abstract

**Introduction:**

Moyamoya disease (MMD) meaning “hazy puff of smoke” in Japanese is a rare chronic cerebrovascular syndrome characterized by progressive stenosis and occlusion of the internal carotid arteries (ICAs) anterior cerebral arteries (ACAs), and middle cerebral arteries (MCAs).

**Methods:**

Our moyamoya patient with severely impaired cognitive and motor functions underwent a specific motor and neuropsychological rehabilitative treatments to assess the recovery of consciousness.

**Results:**

Results obtained showed an improvement of clinical and neuropsychological examination. These findings highlighted the importance of an intensive rehabilitation techniques used in the care of disorders of consciousness patients.

**Conclusions:**

The use of sensory methods provides advantages for the rehabilitation. In fact, in this study, we showed a correlation between sensory stimulation and changes in patient's clinical status.

## 1 INTRODUCTION

Moyamoya disease (MMD) is a rare chronic cerebrovascular syndrome characterized by progressive stenosis and occlusion of internal carotid arteries (ICAs); anterior cerebral arteries, and middle cerebral arteries (MCAs) (Burke et al., 2009; Jefferson, Glosser, Detre, Sinson, & Liebeskind, 2006). The Ministry of Health and Welfare of Japan defined four types of cerebral lesions: hemorrhagic (more frequent in adult population), ischemic (more frequent in childhood population), epileptic, and other causes. Epidemiological studies highlighted that MMD was more frequent in eastern continent such as Japan and Korea and was more prevalent in females than males. There are no differences in sex distribution MMD in Chinese patients. Another epidemiologic different is related to etiology of MMD: in Korea and Japan hemorrhagic events are more common than ischemic stroke (Kim, 2016). High‐field neuroimaging studies provide information about the pathophysiology of cerebrovascular disease (Boulos et al., 2014). In literature, a case of MMD patient who underwent a specific cognitive and motor rehabilitative program was reported. After surgical treatment, patient had a good cognitive and motor recovery. This study showed how rehabilitative program induced neuroplasticity and contributed to a motor and cognitive recovery (Calabrò et al., 2015). We present a case of 31‐year‐old man with moyamoya syndrome and severely impaired cognitive and motor functions. The patient underwent multidisciplinary rehabilitative program in order to demonstrate the importance of sensory and cognitive rehabilitative techniques and to assess the possible recovery of consciousness.

## 2 CASE DESCRIPTION

We report a case of 31‐year‐old right‐handed male patient. He came to our neurorehabilitative care unit after hemorrhagic cerebral event. He presented a severe medical history. He was submitted to craniotomy for decompression of hematoma in fronto‐parietal right regions. He also presented devices for nutrition (PEG) and for spontaneous breath (Tracheal Cannula). Medical management included Baclofen, Ramipril and Levodopa/benserazide cloridrato. All these clinical aspects were compatible with MMD During hospitalization, the patient in our institute, performed a specific motor, logopedic, neuropsychological rehabilitative treatments.

At first, we immediately conducted a neuropsychological and clinical evaluation. Patient was evaluated as a Vegetative State (VS). For this reason, it was impossible to undertake a global neuropsychological evaluation. Patient was awake, but verbal and nonverbal communication were absent. There was no contact with external environment. We used clinical scales to evaluate evolutions of consciousness's state such as: Coma Recovery Scale‐Revised (CRS‐R), Level of Cognitive Functioning (LCF), Disability Rating Scale (DRS), and Glasgow Coma Scale (GCS). He was re‐evaluated at least 1–2 times/week, to monitor conscious improvements (Table 1).

**Table 1:** Periodic evaluation of clinical and consciousness status

SCALES	LCF	CRS‐R	GOS‐E	DRS
TIME 1	2	5	2	26
TIME 2	2	7	2	26
TIME 3	3	8	3	25

**Figure 1:**
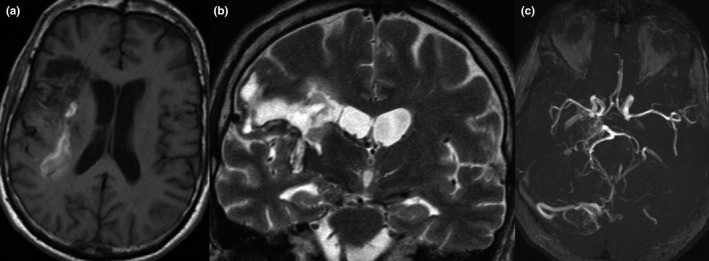
(a) Axial SE T1‐weighted (b) coronal TSE T2‐weighted images Right capsular, fronto‐parietal, and insular lobe hemorrhage. (c)ToF‐3D, MIP reconstructions. The image shows some bilateral nidus of vessels through which arteriovenous shunting occurs

Patient was submitted to magnetic resonance angiography that showed several parenchymal supra‐ and subtentorial brain arteriovenous malformation (AVMs) with "smoke cloud" evident in different cerebral areas: bilateral cerebellum, occipital and posterior temporal areas, and hippocampus. Magnetic Resonance Imaging (MRI) showed intraparenchymal hemorrhagic lesion in right capsular, fronto‐parietal and insular lobe (Figure 1). Electroencephalographic (EEG) evaluation was performed at baseline, and after 4 months: it showed altered electrical brain activity due to the presence of slow polymorphous waves (theta and delta rhythms) prevalent in left hemisphere. In addition, we performed Laser‐Evoked Potentials (LEPs) examination to evaluate sensory and nociceptive system (De Salvo, Caminiti et al., 2015; De Salvo, Naro et al., 2015) and Event‐Related Potentials (ERPs) assessment to explore residual cortical cognitive functions (De Salvo, Caminiti et al., 2015; De Salvo, Naro et al., 2015).

LEPs examination, at baseline time point (T0), showed an increase of latency (300–440 msec) and an amplitude reduction (8,7 µv) of vertex‐complex N2/P2, (more marked in lower limbs), respect to standard value of LEPs (latency: 244–370 msec; amplitude: 16 µv). At follow‐up evaluation (T1), the LEPs highlighted a mild improvement of vertex‐complex N2/P2 to left lower limb.

We used Neurowave System to perform multisensory stimulation and analyze biophysical parameters (De Salvo, Caminiti et al., 2015; De Salvo, Naro et al., 2015). The ERPs recorded, showed moderate improvement between T0–T1. At T0, the P300 wave was absent; while at T1, the P300 wave was present even if the latency and amplitude parameters were significantly altered.

The multidisciplinary team assessed the patient everyday: he was treated for logopedic, physiotherapeutic, and neuropsychological rehabilitation. Physiatrist, in collaboration with neurologists, neuropsychologists, and therapists (logopedists and physiotherapists), planned a rehabilitation program. Members of the patient's family were updated about clinical conditions and the progress of the rehabilitation program every month.

We started rehabilitative treatment 1 month after acute event and was carried out for 6 months (six times a week). Patient was treated two times daily for 60 min to each session. The logopedists, in particular, contributed to tracheostomic cannula with respiratory and phonatory exercises. Physiotherapeutic treatments included passive loosening exercises of upper and lower limbs, manipulation exercises to reduce spasticity, and exercises to improve trunk control. Neuropsychologists used devices to improve the auditory and visual tracking.

At the first cognitive evaluation, we observed that auditory channel was more responsive than visual channel. During rehabilitative process, multisensory devices were used to stimulate visual and auditory channels such as Neurowave System and, Sniffin Stick test to stimulate olfactory way (Bonanno et al., 2017) and tactile stimulation to record different tactile responses (hot, cold, rough, smooth, soft). After 2 months of hospitalization, our patient became more responsive by using visual tracking. Our results confirmed that patient had a moderate recovery of consciousness. In fact, clinical scale scores improved especially between the second and third time point (see Table 1). Patient was assessed as a minimal responder because he showed self‐awareness of the surrounding environment. Although, cognitive and relational profile showed a clinical recovery, motor profile remained unchanged.

## 3 DISCUSSION

The natural history of MMD is not fully understood. There are few studies on disease progression. Early clinical investigations demonstrated that in pediatric patients, who were conservatively treated, functional or intellectual outcome was poor. Adult patients with MMD, who have unilateral lesions, were carefully monitored to control the potential progression to bilateral lesions (Kuroda & Houkin, 2008).

Few studies described MMD cases associated with severe disorders of consciousness. However, motor and cognitive rehabilitative techniques have been extended also to patients with altered consciousness. In particular, the use of sensorial methods provides several advantages for rehabilitation. Studies demonstrated a correlation between sensory stimulation and changes in patient's clinical status (De Salvo, Caminiti et al., 2015; De Salvo, Naro et al., 2015). Several studies described patients with cognitive dysfunctions treated by a specific cognitive rehabilitative program (Calabrò et al., 2015; Coughlin, Miller, & Schuette, 2017; Takagi & Miyamoto, 2015). The neuropsychological and imaging data obtained from the present case showed that, severe cognitive dysfunctions are really disabling in chronic cerebrovascular disorders, including MMD. In this case, we highlighted the importance of neuropsychological periodic assessment to evaluate the improvement of consciousness. In fact, these findings highlighted the importance of intensive rehabilitative techniques (La Gattuta et al., 2018).

This case demonstrated the evidence of a possible recovery of cognitive and motor functions after a MMD. Early‐ and long‐term rehabilitation can affect the outcome in severe brain injury such as MMD. In this case, multidisciplinary programs and intensive rehabilitation were performed. Indeed, it is possible that the long‐term rehabilitation could recover several clinical functions. In addition, a more appropriate model of motor and cognitive multisensory stimulation, programmed by multidisciplinary rehabilitation team, could influence the recovery of consciousness inducing mechanisms of neuronal plasticity.

## CONFLICT OF INTEREST

The authors declare that they have no conflict of interest.
